# Determinants of Plasma 25-Hydroxyvitamin D Concentrations among Breast Cancer Survivors in Korea

**DOI:** 10.3390/nu10030380

**Published:** 2018-03-20

**Authors:** Woo-Kyoung Shin, Zisun Kim, Hyun Jo Youn, Jihyoung Cho, Jung Eun Lee

**Affiliations:** 1 Research Institute of Human Ecology, Seoul National University, Seoul 08826, Korea; shiningwk@gmail.com; 2 Department of Surgery, Soonchunhyang University Bucheon Hospital, Bucheon 14584, Korea; sk4091@hanmail.net; 3 Department of Surgery, Chonbuk National University Medical School, Jeonju 54907, Korea; yhj0903@jbnu.ac.kr; 4 Breast Endocrine Division, Department of Surgery, Keimyung University School of Medicine, Daegu 41941, Korea; chojh0404@gmail.com; 5 Department of Food and Nutrition, Seoul National University, Seoul 08826, Korea

**Keywords:** vitamin D, 25-hydroxyvitamin D, vitamin D insufficiency, breast cancer survivors

## Abstract

We identified demographic, lifestyle, and clinical factors associated with vitamin D status among breast cancer survivors. The vitamin D prediction model may be a useful surrogate of circulating 25-hydroxvitamin D (25(OH)D) concentrations when this measure was not available. We included a total of 216 Korean breast cancer survivors aged 21–79 years who had been diagnosed with stage I to III primary breast cancer and had breast cancer surgery at least 6 months before enrolment. We used linear and logistic regressions to identify determinants for the plasma 25(OH)D concentrations and vitamin D insufficiency (plasma 25(OH)D concentration < 50 nmol/L). We observed that 48.85% of breast cancer survivors had a plasma 25(OH)D concentration less than 50 nmol/L. We identified the following determinants for plasma 25(OH)D concentrations: time since diagnosis (β = −0.005 for 1 month increment), supplementary vitamin D intake (β = 0.06 for 10 μg/day increment), season of the blood draw (β = 0.35 for summer; β = 0.32 for fall; β = 0.26 for winter vs. spring), smoking status (β = 0.28 for former vs. never), use of any supplement (β = −0.35 for non-use vs. use), and the parity number (β = −0.30 for three or more vs. one) were associated with the plasma 25(OH)D concentrations. In addition to the aforementioned variables, body mass index (BMI) was associated with the prevalence of vitamin D insufficiency. We identified the determinants for the plasma 25(OH)D concentrations among Korean breast cancer survivors. Future studies are needed to investigate the role of vitamin D in the progression of breast cancer among Korean breast cancer survivors.

## 1. Introduction

Vitamin D from diet and supplements and from synthesis in the skin is first metabolized to 25-hydroxyvitamin D (25(OH)D) in the liver and then further metabolized to 1,25-dihydroxyvitamin D (1,25(OH)2D) by 1-α-hydroxylase in the kidney and other tissues. The actions of 1,25(OH)2D are receptor mediated, and vitamin D receptors are present in normal and malignant breast tissues [1]. Increasing the vitamin D concentrations has been suggested to decrease the risk of breast cancer [2,3].

Carcinogenesis-related functions of vitamin D include an increase in cellular differentiation, T-cell mediated immunity, cell-cycle arrest, and apoptosis and a decrease in proliferation, invasiveness, and metastasis [1,4,5,6,7].

Given that the vitamin D status has been suggested to be important for cancer prevention in several experimental and epidemiologic studies, several studies have assessed vitamin D status and identified its modifiable factors. Plasma 25(OH)D, the primary circulating form of vitamin D, is considered an appropriate biomarker of the vitamin D status in humans [8]. The vitamin D status in the circulation is determined by exogenous sources from dietary and supplemental intake, endogenous production through synthesis in the skin, and the activities of vitamin D metabolic enzymes [9,10]. However, the 25(OH)D concentration is greatly dependent on the season of the blood draw, and the measurement of the 25(OH)D concentration, which requires the availability of a blood sample and the cost of a laboratory assay, limiting its feasibility in long-term exposure studies and in large-scale epidemiological studies. For this reason, the vitamin D prediction model may be a useful tool for surrogates of circulating 25(OH)D concentrations in research on breast cancer patients for which biomarkers are not available. Also, understanding the determinants of the 25(OH)D concentrations may be important because some can be modified at the individual level and can be targeted as part of public health initiatives.

Several epidemiological studies have reported on the potential determinants of vitamin D status among populations that did not have cancers. In the Nurses’ Health Study, race, UV-B flux, dietary vitamin D, supplementary vitamin D, body mass index (BMI), physical activity, postmenopausal hormone use, alcohol intake, and season of the blood draw were major factors related to circulating 25(OH)D concentrations [11]. The Physical Activity and Nutrition in Children (PANIC) Study found that age, physical activity, milk product intake, vitamin D intake from supplement (girls), household income (boys), and sunscreen use (boys) were associated with circulating 25(OH)D concentrations. [12]. In the Black Women’s Health Study, age, season of the blood draw, UV-B flux, supplement vitamin D, dietary vitamin D, BMI, postmenopausal hormone use, vigorous physical activity, alcohol consumption, smoking, recent use of oral contraceptives, and use of oral contraceptives for more than 10 years [13] were associated with circulating vitamin D concentrations. Several previous Asian studies reported that the frequency of fish and egg consumption, outdoor work, having a history of resection of any part of the gastrointestinal tract, age, total hours of sunshine, weight, and suntan in the past year were associated with 25(OH)D concentrations [14,15,16,17]. However, there are relatively few studies examining the factors associated with circulating vitamin D concentrations among cancer survivors whose health behaviours may differ from the general population such as dietary intake, supplement use, physical activity, and smoking habit [18,19,20,21]. Additionally, the factors related to tumour growth and treatment including cancer stage, grade, tumor stage, lymph node status, types of therapy, epithelial proliferative activity, and intrinsic subtype may be related to the vitamin D status among cancer survivors [22,23,24].

In this study, we aimed to develop a predicted 25(OH)D score and identify the demographic, lifestyle, and clinical factors associated with vitamin D status among breast cancer survivors.

## 2. Methods

### 2.1. Study Participants

We included a total of 229 women aged 21 to 79 years who were enrolled between June 2015 and January 2017 at three large hospitals and whose plasma 25(OH)D concentrations were measured. The participants were diagnosed with stage I to III primary breast cancer according to the American Joint Committee on Cancer (AJCC) criteria and had breast cancer surgery at least 6 months before enrollment. The median survival time since diagnosis of participants was 25 months (interquartile range: 18–48 months). We excluded women with a 25(OH)D concentration that was less than the detection limit (7.5 nmol/L) (*n* = 4), other cancers before the breast cancer diagnosis (*n* = 3), any cancer after diagnosis but before enrollment (*n* = 3), and no medical records (*n* = 3). We also excluded women whose plasma 25(OH)D concentration was beyond ±3 standard deviations from the mean (*n* = 3). As a result, our analysis included a sample of 216 breast cancer survivors. When we conducted a sensitivity analysis including the 3 participants whose plasma 25(OH)D concentrations were beyond ±3 standard deviations from the mean, the results were similar. The institutional review board of each hospital approved all the procedures for this study (Soonchunhyang University Bucheon Hospital: SCHBC 2014-12-004-001; Chonbuk National University Hospital: CUH 2014-05-002-005; Keimyung University Dongsan Medical Center: DSMC 2015-03-026-003). Written informed consent was obtained from all participants.

### 2.2. Demographic and Clinical Factors

We asked the participants about their demographic, socioeconomic, and lifestyle factors at enrollment. The demographic questionnaire captured the survivors’ use of any supplement (yes/no), type, dose, and duration of supplementation, education level, marital status, smoking status (never/former/current), dose and duration of smoking, and the frequency and amount of alcohol intake(never/ever). We asked participants about the type, duration and frequency of each physical activity. As additional questions, participants were asked to list types of exercises that they commonly engaged in and their duration and frequency. A metabolic equivalent (MET) value was assigned to each activity reported according to the Compendium of Physical Activities [25]. To assess daily sun exposure, we collected information on daily sun exposure from 10:00 a.m. to 4:00 p.m. for weekdays and the weekend. We asked participants about hours of daily sun exposure (less than 5 min/5–15 min/15–60 min/1–3 h/more than 3 h). We calculated the average minutes of daily sun exposure. We obtained information on the skin phototype sensitivity including skin phototype change during an hour in the summer sun without sunscreen after several months of not being in the sun (blistering sunburn/a sunburn without blisters/a mild sunburn that becomes a tan/a tan with no sunburn/no change in skin color), the formation of blisters, and the use of a hat or clothes in the summer. We obtained information on the time since surgery, AJCC stage at diagnosis, menopausal status at diagnosis and age, height, and weight at diagnosis through the medical records.

### 2.3. Dietary Assessment

To assess dietary intake, we collected information on the foods and amounts consumed using 3-day dietary records for each participant. Participants were asked to write down every food and dish that they consumed on three non-consecutive days, including two weekdays and one weekend day. Following the protocol, research dietitians reviewed the unclear descriptions, errors, omissions, or questionable entries; and asked the participants to clarify entries as necessary. Vitamin D and calcium intakes were calculated from both food and supplemental sources. For the nutrient database of the 3-day dietary records, we used CAN-Pro 5.0 (Computer Aided Nutritional Analysis Program 5.0; The Korean Nutrition Society, Seoul, Korea). Vitamin D and calcium intakes were calculated after energy adjustment by the residual method [26].

### 2.4. Measurement of Plasma 25(OH)D Concentration

After the blood draw at enrollment, blood samples were moved to the laboratory within 24 h and centrifuged. Plasma 25(OH)D concentrations were determined by the electrochemiluminescence immunoassay (CVs ranged from 5.83% to 8.28%) (Seegene Medical Foundation, Seoul, Korea).

### 2.5. Statistical Analyses

Model selection was performed in a training sample (80% of the total participants, *n* = 178) to identify the optional determinants in the generalized linear model for 25(OH)D using PROC GLMSELECT in SAS. We used the test sample (20% of the total participants, *n* = 38) from the participants with 25(OH)D measurements to validate the prediction model. 

Using the training sample, we fitted the linear regression model to predict the measured plasma 25(OH)D (nmol/L) based on known or potential determinants. Age at diagnosis (years) was included as an independent variable in all the models. The following potential determinants were included in the analyses: total energy intake (kcal/day, continuous), vitamin D intake from food (μg/day, continuous), calcium intake from food (mg/day, continuous), vitamin D intake from supplement (μg/day, continuous), calcium intake from supplement (mg/day, continuous), BMI (kg/m^2^, continuous), physical activity (MET-hours/week, continuous), time since diagnosis (month, continuous), daily sun exposure (min, continuous), the amount of sunshine based on residence (hours per year, continuous), season of the blood draw (spring, summer, fall, winter), smoking status (never, former), alcohol intake (never, ever), use of any supplement (yes, no), menopausal status (premenopause, postmenopause), hormonetherapy (yes, no), chemotherapy (yes, no), radiation therapy (yes, no), ER status (−, +), hair color (black, light brown, brown, dark brown), skin phototype change during an hour in the summer sun without sunscreen after several months of not being in the sun (blistering sunburn, a sunburn without blisters, a mild sunburn that becomes a tan, tan with no sunburn, no change in skin phototype color), use of a hat or clothes in the summer (never, rarely, sometimes, often, always), the parity number (none, 1, 2, 3 or more), and the centre enrolled in this study.

We used a stepwise linear regression to identify determinants of vitamin D status. The significance level for entering (SLENTRY) and the significance level for staying (SLSTAY) were 0.1. The final multivariable prediction model includes statistically significant determinants plus the age at diagnosis. To examine whether the assumptions of the linear regression were met, we performed normality and homoscedasticity tests. Because the distribution of the plasma 25(OH)D concentrations was skewed, we log-transformed it and found that the assumptions were satisfied. We conducted two sensitivity analysis. Because the participants in centre 1 had a higher proportion of vitamin D supplement use and a higher proportion of past smokers compared to those in the other two centres, we conducted the first sensitivity analysis by limiting our analysis to patients who never smokers. In the second sensitivity analysis, we adjusted for centre using the residual model. To remove the variation in the centres, we obtained residuals from the linear regression of each natural log-transformed plasma 25(OH)D controlling for the centre. The mean natural log-transformed plasma 25(OH)D concentration was added back to the residual.

Based on the regression coefficients for each variable in the prediction model, we calculated a predicted 25(OH)D score for each individual in the test samples. For validation, we compared the predicted 25(OH)D with the actual plasma 25(OH)D measurements in the test sample. When we compared predicted 25(OH)D with actual plasma 25(OH)D measurements in the test sample, we exponentially log-transformed the 25(OH)D concentrations. Spearman correlation coefficients were calculated to assess the agreement between the predicted score and the actual 25(OH)D concentrations. We examined the actual plasma 25(OH)D measurements according to the quintiles of the predicted 25(OH)D score. We also evaluated the agreement between the predicted 25(OH)D and the actual plasma 25(OH)D measurements using the Bland-Altman analysis [27]. 

To select the determinants of vitamin D insufficiency (<50 nmol/L) [28], we used a stepwise selection procedure within the logistic regression model. We used a stepwise logistic regression to identify determinants of the vitamin D status. The significance level for entering (SLENTRY) and the significance level for stay (SLSTAY) values were 0.1. Because we had missing variables for energy intake (*n* = 1), supplementary vitamin D and calcium intake (*n* = 3), use of any supplement (*n* = 6), smoking status (*n* = 22), alcohol intake (*n* = 2), the parity number (*n* = 12), and the questionnaire about lifestyle including natural hair color (*n* = 4), skin phototype change during an hour in the summer sun without sunscreen after several months of not being in the sun (*n* = 7), and the use of a hat or clothes in the summer (*n* = 1), we used multiple imputation for continuous variables, but used single imputation for the categorical variables, assigning missing individuals to the most common category.

The statistical software package SAS for Windows version 9.4 (SAS Institute, Cary, NC, USA) was used for all statistical analysis. 

## 3. Results

**Figure 1:**
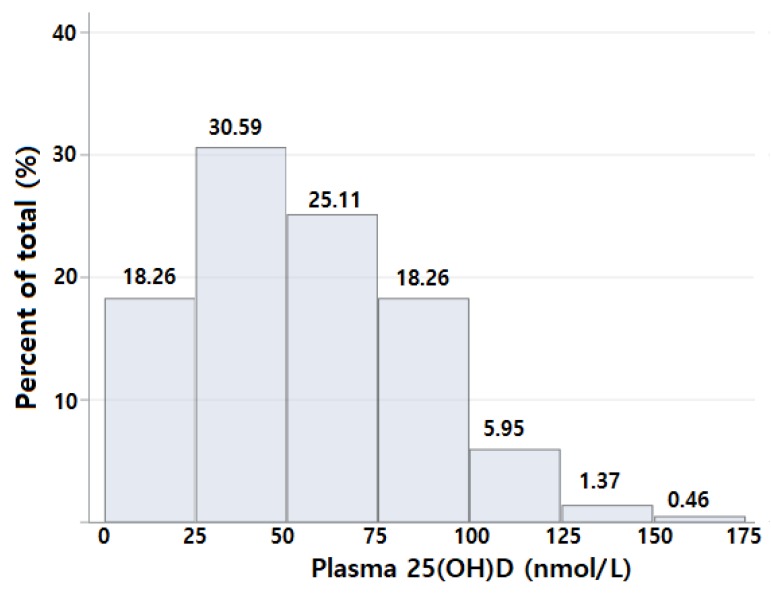
Measured plasma 25(OH)D (nmol/L) among breast cancer survivors in Korea.

Figure 1 shows the distribution of the plasma 25(OH)D concentrations. Among the breast cancer survivors, 30.59% had 25–50 nmol/L, and 25.11% had 50–75 nmol/L of plasma 25(OH)D. The mean value (SD) of the plasma 25(OH)D concentrations was 54.14 (30.88) nmol/L among all the participants, 59.42 ± 30.21 nmol/L among the users of any supplement (71.23%), and 39.14 ± 27.90 nmol/L among the non-users of any supplement (26.03%). Table 1 presents the demographic, dietary and clinical factors according to the plasma 25(OH)D concentrations. Participants with vitamin D insufficiency (<50 nmol/L) were more likely to have a higher BMI and to consume lower amounts of total, dietary and supplementary vitamin D and were less likely to use any supplements compared to those with ≥50 nmol/L of 25(OH)D. Participants in centres 2 and 3 had a higher proportion of vitamin D insufficiency than those in centre 1. We also compared the characteristics of the participants across centres (Supplementary Materials Table S1).

We identified the following determinants of the plasma 25(OH)D concentrations in the training sample; time since diagnosis, dietary and supplementary vitamin D intake, season of the blood draw, smoking status, use of any supplement, the parity number and centre (Table 2). When we included all of these determinants in the multivariate linear regression models (Adjusted *R*^2^ = 0.33); we identified that time since diagnosis (β = −0.005 for 1 month increment), supplementary vitamin D intake (β = 0.06 for 10 μg/day increment), season of the blood draw (β = 0.35 for summer; β = 0.32 for fall; β = 0.26 for winter vs. spring), smoking status (β = 0.28 for former vs. never), use of any supplement (β = −0.35 for non-use vs. use), the parity number (β = −0.30 for three or more vs. one), and the centre (β = −0.30 for centre 2 vs. 1; β = −0.36 for centre 3 vs. 1) were associated with the plasma 25(OH)D concentrations (Table 2).

**Figure 2:**
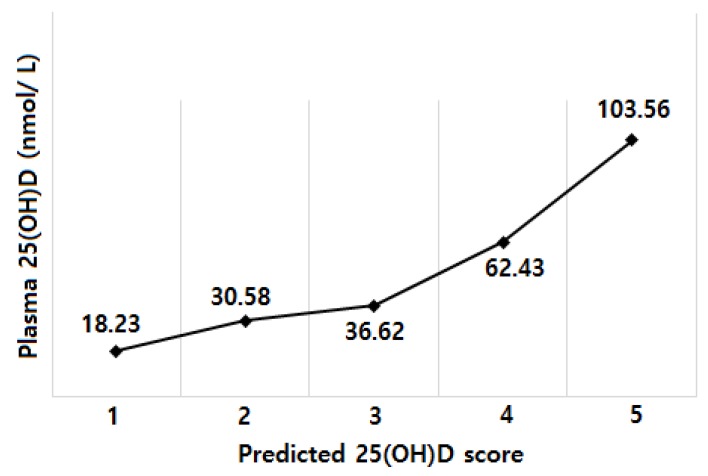
Mean actual 25(OH)D concentrations by quintile of predicted 25(OH)D score in the validation sample.

We calculated the predicted 25(OH)D scores for each participant based on the intercept and the regression coefficients in the test sample. The Spearman correlation coefficients between the predicted score and the actual 25(OH)D concentrations were 0.54 (*p* < 0.001) in the test sample. When we calculated the mean value of the actual 25(OH)D concentrations according to the quintiles of the predicted score and evaluated the agreement using the Bland-Altman analysis, the actual plasma 25(OH)D concentrations generally rose with increasing quintiles of the predicted 25(OH)D (Figure 2), and a total of 92.11% (35 of 38) of the values lie within 2 SD of the mean difference. 

When we limited the analysis to patients who were never smokers, we identified the following determinants of the plasma 25(OH)D concentrations (Adjusted *R*^2^ = 0.34): time since diagnosis (β = −0.004 for a 1 month increment), supplementary vitamin D intake (β = 0.11 for 10 μg/day increment), use of any supplement (β = −0.38 for nonuse vs. use), menopausal status (β = −0.38 for premenopausal vs. postmenopausal), physical activity (β = 0.25 for 3rd tertile vs. 1st tertile), the parity number (β = −0.34 three more vs. one), and centre (β = −0.31 centre 3 vs. centre 1). When we calculated the predicted 25(OH)D scores among never smokers, we found that the correlation between the predicted score and actual 25(OH)D concentrations was 0.45 (*p* < 0.001) in the test sample.

We explored the factors associated with low 25(OH)D concentrations (<50 nmol/L) in the multivariate logistic regression models (Table 3). The associations between the significant determinants and low 25(OH)D concentrations (<50 nmol/L) were as follows; supplementary vitamin D intake (Odds Ratio (OR) 0.44; 95% Confidence Interval (CI) 0.29–0.66 for 10 µg/day increment), BMI (Odds Ratio (OR) 1.16; 95% Confidence Interval (CI) 1.03–1.31 for 1 kg/m^2^ increment), time since diagnosis (OR 1.02; 95% CI 1.01–1.04 for 1 month increment), smoking status (OR 0.09; 95% CI 0.02–0.38) for former vs. never), and use of any supplement (OR 2.88; 95% CI 1.24–6.65 for no vs. yes). We presented ORs (95% CIs) for all the factors that we examined in a stepwise procedure of logistic regression models (Supplementary Materials Table S2).

When we limited the analysis to never smokers, supplementary vitamin D intake (OR 0.40; 95% CI 0.24–0.67 for 10 μg/day increment), BMI (OR 1.22; 95% CI 1.06–1.41 for 1 kg/m^2^ increment), time since diagnosis (OR 1.02; 95% CI 1.01–1.04 for 1 month increment), use of any supplement (OR 3.11; 95% CI 1.17–8.26 for no vs. yes), and centre (OR 7.33; 95% CI 2.53–1.29 for centre 2 vs. centre 1; OR 7.81; 95% CI 2.64–23.04 for centre 3 vs. centre 1) were associated with low 25(OH)D concentrations.

## 4. Discussion

In our study of breast cancer survivors, we identified the determinants for the plasma 25(OH)D concentrations among breast cancer survivors. In the linear regression models, we found that the use of any supplement was associated with the plasma 25(OH)D concentrations. Additionally, time since diagnosis, supplementary vitamin D intake, season of the blood draw, smoking status, and the parity number were significantly associated with the 25(OH)D concentrations. We validated this predicted vitamin D score in a test sample. When we examined the factors associated with vitamin D insufficiency (less than 50 nmol/L), we found that supplementary vitamin D intake, BMI, time since diagnosis, smoking status, and use of any supplement were significant determinants.

In agreement with our study, several previous studies have reported similar results. The season of the blood draw was associated with circulating 25(OH)D concentrations [29,30,31]. Circulating 25(OH)D concentrations were the highest in the late summer and early autumn and lowest in late winter and early spring [13,32]. Several studies found that former smokers had higher 25(OH)D concentrations [33,34], which is consistent with our research. However, other studies found that current smoking was associated with lower circulating 25(OH)D concentrations compared with never smoking [13,33,35]; however, there was no association between smoking status and the 25(OH)D concentrations in other studies [36,37,38]. We were unable to compare the vitamin D status of current smokers with that of never smoker because we had no current smokers among the breast cancer survivors in our study. In our study, the participants in centre 1 had a higher proportion of vitamin D supplement use and a higher proportion of past smokers compared to those in the other two centres and thus this may be reflected in our results. Increased the dietary and supplementary vitamin D intake was associated with increased concentrations of 25(OH)D among cancer survivors [30]. A high parity was an independent determinant of low circulating 25(OH)D concentrations [39,40,41].

Obesity was associated with low circulating 25(OH)D concentrations or vitamin D insufficiency in various epidemiologic studies [11,29,33,42,43]. A potential explanation was a greater dilution of vitamin D, a fat-soluble vitamin, in the adipose tissue, which may result in reduced circulating levels [44,45]. In addition, overweight or obese people have a tendency to be less often exposed to sunlight because of lower exercise levels and decreased mobility [32]. The characteristics associated with sun exposure-related behaviour such as wearing of hats or long sleeves/pants, use of sunscreen, and use of a tanning bed were considered to be the factors explaining the variation in the blood 25(OH)D concentrations [11,12,37,42,46,47]. A tendency to burn after unprotected sun exposure was associated with vitamin D insufficiency [12,48,49]. In our study, the use of a hat or clothes in the summer was marginally non-significant (*p* value = 0.11); therefore, the final model did not include this factor. However, it warrants further studies.

Our model explained 33% of the between-participant variance in breast cancer survivors. Previous model prediction studies reported a range between 21% and 42% of variation in the training sample [11,29,42,50,51,52]. Moreover, the correlation coefficient of 0.54 (*p* < 0.0001) in our test sample was similar to the correlation reported by the previously prediction models [11,29,42,52].

The present study has some limitations. First, we had only one measure of plasma 25(OH)D concentration, and it is unclear how these concentrations may vary within individuals over time or if only one-time measurement of the 25(OH)D concentration is adequate. However, a single measure of vitamin D metabolites has shown to be a practical marker of long-term vitamin D status 3–4 years apart [53]. Although the predicted 25(OH)D value may not be accurate, we found that the predicted 25(OH)D concentrations may be a useful tool for ranking the individuals based on circulating vitamin D concentrations. A comparison of the low vs. high 25(OH)D estimates may be applicable to examine the association between vitamin D status and disease in epidemiological studies [11]. Second, the sampling for this study was not random, which limits our ability to generalize our results to all breast cancer survivors in Korea. Third, we did not include information on the genetic variants in the prediction model. However, a previous study found that adding genetic variants in the prediction model of 25(OH)D concentrations explained an additional <5% of the overall variation in the 25(OH)D concentrations [54]. The season of the blood draw and use of a vitamin D supplement made a substantial difference in explaining the variation in the 25(OH)D concentrations compared to the genetic determinants [34]. Fourth, our prediction model had relevant unexplained variability, which probably can be explained by errors in the measurement of the variables and the unmeasured determinants of the vitamin D status such as actual UV radiation. However, we obtained detailed information on sun exposure including residence for the longest time, sun exposure for 2 years according to the season, the frequency of sun exposure from 10:00 to 16:00, skin phototype sensitivity including skin phototype change during an hour in the summer sun without sunscreen after several months of not being in the sun, the formation of blisters, and the use of a hat or clothes in the summer.

Our study has some strengths. This study is the first study to identify the factors associated with circulating 25(OH)D concentrations among breast cancer survivors in Korea. The predicted vitamin D scores may be a practical alternative for studying associations between vitamin D status and disease when a biomarker is not available. We assessed the participants’ diet using 3-day dietary records, which may provide an estimate of the habitual intake for individuals.

In this study on Korean breast cancer survivors, we identified the factors associated with either circulating 25(OH)D concentrations or vitamin D insufficiency (less than 50 nmol/L). We found that time since diagnosis, supplementary vitamin D intake, season of the blood draw, smoking status, use of any supplement, and the number of parity were associated with plasma 25(OH)D concentrations. For the prevalence of vitamin D insufficiency (less than 50 nmol/L), we identified supplementary vitamin D intake, BMI, time since diagnosis, smoking status, and use of any supplement. Future studies are needed to investigate the role of vitamin D in the progression of breast cancer among Korean breast cancer survivors.

## Figures and Tables

**Table 1:** Characteristics of study participants according to plasma 25(OH)D concentrations.

Variables	All (*n* = 219)	Plasma 25(OH)D Concentrations	
		<50 nmol/L (*n* = 107)	≥50 nmol/L (*n* = 112)
Age at diagnosis (years)	48 (43–53)	48 (42–53)	48 (44–52)
Body mass index at diagnosis (kg/m^2^)	23.37 (21.63–25.74)	24.14 (21.91–26.37)	25.29 (21.03–25.29)
Energy intake (kcal/day)	1695.25 (1410.58–2039.44)	1684.84 (1428.35–1979.86)	1737.09 (1379.82–2055.63)
Physical activity (MET-hours/week)	49.95 (11.70–49.95)	26.20 (10.00–47.15)	28.41 (15.90–52.35)
Total vitamin D intake (µg/day)	5.01 (0.06–15.56)	2.21 (0.00–7.00)	10.01 (1.76–24.05)
Supplementary vitamin D intake (µg/day)	2.5 (0.00–10.00)	0.00 (0.00–5.00)	10.00 (0.00–25.00)
Use of any supplement			
Yes	156 (71.23)	62 (57.94)	94 (83.93)
No	57 (26.03)	42 (39.25)	15 (13.39)
Time since surgery			
6 month–<1 year	2 (0.91)	1 (0.93)	1 (0.89)
1 year–<3 years	144 (65.75)	64 (59.81)	80 (71.43)
3 year–<5years	35 (15.98)	16 (14.95)	19 (16.96)
5 years and more	38 (17.35)	26 (24.30)	12 (10.71)
AJCC ^a^ stage			
I	102 (46.58)	51 (47.66)	51 (45.54)
II	86 (39.27)	39 (36.45)	47 (41.96)
III	31 (14.16)	17 (15.89)	14 (12.50)
Season of the blood draw			
Spring	48 (21.92)	28 (26.17)	20 (17.86)
Summer	64 (29.22)	32 (29.91)	32 (28.57)
Fall	58 (26.48)	24 (22.43)	34 (30.36)
Winter	49 (22.37)	23 (21.50)	26 (21.50)
Menopausal status at the diagnosis			
Yes	151 (68.95)	72 (67.29)	79 (70.54)
No	68 (31.05)	35 (32.71)	33 (29.46)
Alcohol intake			
Never	94 (43.32)	51 (48.11)	43 (38.74)
Ever	123 (56.68)	55 (51.89)	68 (61.26)
Smoking status			
Never	175 (79.91)	91 (85.05)	84 (75.00)
Former ^b^	22 (10.05)	3 (2.80)	19 (16.96)
Education level			
High school or less	149 (68.35)	74 (69.81)	75 (66.96)
College or more	69 (31.65)	32 (30.19)	37 (33.04)
Marital status			
Married or cohabitation	172 (79.26)	84 (80.00)	88 (78.57)
Unmarried or divorced or widowed	45 (20.74)	21 (20.00)	24 (21.43)
Parity number			
None	37 (16.89)	14 (13.08)	23 (20.54)
1	128 (58.45)	65 (60.75)	63 (56.25)
2	42 (19.18)	25 (23.36)	17 (15.18)
3 and more	10 (4.57)	2 (1.87)	8 (1.87)
Centre			
1	79 (36.07)	17 (15.89)	62 (55.36)
2	70 (31.96)	41 (38.32)	29 (25.89)
3	70 (31.96)	49 (45.79)	21 (18.75)

Total number of participants was not equal to total participants (*n* = 219) because some did not provide information; Continuous variables are reported as median value (interquartile range) and categorical variables are reported as the number of participants. (%); ^a^ AJCC: American Joint Committee on Cancer; ^b^ Only past smoker was included because no one smoked at enrollment.

**Table 2:** Determinants of plasma 25(OH)D concentrations in the training sample.

Determinants ^a^	Difference in 25(OH)D (nmol/L; β)	*p* Value
Age at the diagnosis (per 1 year)	−0.001	0.83
Time since diagnosis (per 1 month)	−0.005	0.01
Supplementary vitamin D intake (per 10 ug/day)	0.06	0.10
Season of the blood draw		
Spring (referent)	0	
Summer	0.35	0.02
Fall	0.32	0.005
Winter	0.26	0.03
Smoking status		
Never (referent)	0	
Former ^b^	0.28	0.03
Use of any supplement		
Yes (referent)	0	
No	−0.35	0.0007
Parity number		
None	−0.10	0.63
1 (referent)	0	
2	−0.04	0.70
3 or more	−0.30	0.02
Centre		
1 (referent)	0	
2	−0.30	0.02
3	−0.36	0.002

^a^ Variables selected by a stepwise procedure in the linear regression model (Adjusted *R*^2^ = 0.33); ^b^ Only past smoker was included because no one smoked at enrollment.

**Table 3:** Odds ratio (OR) and 95% confidence interval (CI) for the association between selected factors and plasma 25(OH)D status (<50 nmol/L) among the breast cancer survivors.

Determinants ^a^	Number of Case/Total	OR (95% CI)
Age at the diagnosis (per 1 year)	107/219	0.99 (0.94, 1.04)
Supplementary vitamin D intake (per 10 µg/day)	107/219	0.44 (0.29, 0.66)
Body mass index at the diagnosis (per 1 kg/m^2^)	107/219	1.16 (1.03, 1.31)
Time since diagnosis (per 1 month)	107/219	1.02 (1.01, 1.04)
Smoking status		
Never (referent)	91/175	1.00
Former ^b^	3/22	0.09 (0.02, 0.38)
Use of any supplement		
Yes (referent)	62/156	1.00
No	42/57	2.88 (1.24, 6.65)

^a^ Variables selected by a stepwise procedure in the logistic regression model; ^b^ Only past smoker was included because no one smoked at enrollment.

## Supplementary Materials

The following are available online at http://www.mdpi.com/2072-6643/10/3/380/s1. Table S1: Characteristics of study participants according to centre. Table S2: Odds ratio (OR) and 95% confidence interval (CI) for the association between factors and plasma 25(OH)D status (<50 nmol/L) among breast cancer survivors.
