# Supplementary material for: Determinants of Plasma 25-Hydroxyvitamin D Concentrations among Breast Cancer Survivors in Korea
Source: Nutrients. 2018 Mar 20;10(3):380. doi: 10.3390/nu10030380 (PMC5872798; doi:10.3390/nu10030380)
Supplement: Supplementary file 1 [file nutrients-10-00380-s001.pdf]

Table S1. Characteristics of study participants according to centre.

**Variables**	**All (*n* = 219)**	**Centre**		
		1 (*n* = 79)	2 (*n* = 70)	3 (*n* = 70)
Plasma 25(OH)D (nmol/L)	50.77 (29.30–75.48)	67.59 (51.94–81.24)	37.46 (28.73–62.57)	32.85 (21.74–55.61)
Age at diagnosis (years)	48.00 (43.00–53.00)	49.00 (45.00–53.00)	47.00 (44.00–53.00)	46.50 (41.00–52.00)
Body mass index at the diagnosis (kg/m^2)	23.37 (21.63–25.74)	23.22 (21.66–25.71)	22.59 (21.36–25.67)	23.94 (21.74–25.79)
Energy intake (kcal/day)	1695.25 (1410.58–2039.44)	1742.43 (1395.87–2052.45)	1667.10 (1372.56–1927.59)	1693.48 (1417.25–2078.25)
Physical activity (MET hours per week)	49.95 (11.70–49.95)	29.00 (0.00–56.20)	30.12 (16.57–52.00)	21.75 (9.33–39.75)
Total vitamin D intake (μg/day)	5.01 (0.06–15.56)	12.66 (12.21–27.63)	3.41 (0.00–12.21)	2.62 (0.00–7.24)
Supplementary vitamin D intake (μg/day)	2.5 (0.00–10.00)	10.00 (0.00–25.00)	0.00 (0.00–10.00)	0.00 (0.00–10.00)
Dietary supplement use				
Yes	156 (71.23)	67 (84.81)	44 (62.86)	45 (64.29)
No	57 (26.03)	11 (13.92)	24 (34.29)	22 (31.43)
Time since surgery				
6 month–<1 year	2 (0.91)	1 (1.27)	1 (1.43)	0
1 year–<3 years	144 (65.75)	49 (62.03)	61 (87.14)	34 (48.57)
3 years–<5years	35 (15.98)	16 (20.25)	8 (11.43)	11 (15.71)
5 years and more	38 (17.35)	13 (16.46)	0	25 (35.71)
AJCC [a] stage				
I	102 (46.58)	27 (34.18)	46 (65.71)	29 (41.43)
II	86 (39.27)	38 (48.10)	17 (24.29)	31 (44.29)
III	31 (14.16)	14 (17.72)	7 (10.00)	10 (14.29)
Season of the blood draw				
Spring	48 (21.92)	25 (31.65)	0	23 (32.86)
Summer	64 (29.22)	11 (13.92)	52 (74.29)	1 (1.43)
Fall	58 (26.48)	29 (36.71)	18 (25.71)	11 (15.71)

winter	49 (22.37)	14 (17.72)	0	35 (50.00)
Menopausal status at the diagnosis				
Yes	151 (68.95)	53 (67.09)	47 (67.14)	51 (72.86)
No	68 (31.05)	26 (32.91)	23 (32.86)	19 (27.14)
Alcohol intake				
Never	94 (43.32)	28 (36.36)	35 (50.00)	31 (44.29)
Ever	123 (56.68)	49 (63.64)	35 (50.00)	39 (55.71)
Smoking status				
Never	175 (79.91)	52 (65.82)	64 (91.43)	59 (84.29)
Former [b]	22 (10..05)	15 (18.99)	2 (2.86)	5 (7.14)
Education level				
High school or less	149 (68.35)	57 (72.15)	48 (68.57)	44 (63.77)
College or more	69 (31.65)	22 (27.85)	22 (31.43)	25 (36.23)
Marital status				
Married or cohabitation	172 (79.26)	64 (82.05)	58 (82.86)	50 (72.46)
Unmarried or divorced or widowed	45 (20.74)	14 (17.95)	12 (17.14)	19 (27.54)
Parity number				
None	37 (16.89)	3 (3.80)	4 (5.71)	3 (4.29)
1	128 (58.45)	16 (20.25)	9 (12.86)	12 (17.14)
2	42 (19.18)	46 (58.23)	41 (58.57)	41 (58.57)
3 and more	10 (4.57)	13 (16.46)	16 (22.86)	13 (18.57)

Continuous variables are reported as median value (interquartile range) and categorical variables are reported as number of participants. (%); [a]AJCC: American Joint Committee on Cancer; [b]Only past smoker was included because no one smoked at enrollment.

Table S2. Odds ratio (OR) and 95% confidence interval (CI) for the association between factors and plasma 25(OH)D status (< 50 nmol/ml) among breast cancer survivors.

Variables [a]	Number of case/total	OR (95% CI)
Age (per 1 year)	107/219	0.96 (0.88 , 1.04)
Time since diagnosis (per 1 moth)	107/219	1.03 (1.01 , 1.05)
Total energy intake (per 1 kcal/day)	107/219	1.00 (1.00 , 1.00)
Supplementary vitamin D intake (per 10 μg/day)	107/219	0.53 (0.25 , 1.12)
Supplementary calcium intake (per 10 mg/day)	107/219	1.00 (0.97 , 1.03)
Body mass index	107/219	1.20 (1.03 , 1.39)
Physical activity (per 1 met-hours/day)	107/219	1.00 (0.99 , 1.01)
Daily sun exposure (per 1 minutes/day)	107/219	1.00 (1.00 , 1.00)
Season		
Spring (referent)	28/48	1.00
Summer	32/64	0.21 (0.04 , 1.12)
Fall	24/58	0.29 (0.08 , 1.12)
winter	23/49	0.40 (0.11 , 1.50)
Smoking status		
Never (referent)	91/175	1.00
Former [b]	3/22	0.08 (0.01 , 0.52)
Alcohol intake		
Never drinker (referent)	56/124	1.00
Ever drinker	51/92	1.10 (0.47 , 2.60)
Supplement use		
Yes (referent)	62/156	1.00
No	42/57	4.05 (1.41 , 11.62)
Postmenopausal status		
Yes (referent)	72/151	1.00
No	35/68	1.92 (0.61 , 6.01)

Radiation therapy		
Yes (referent)	86/166	1.00
No	21/50	1.23 (0.46 , 3.28)
Chemo therapy		
Yes (referent)	83/175	1.00
No	24/41	0.86 (0.30 , 2.45)
Hormone therapy		
Yes (referent)	76/166	1.00
No	29/50	0.27 (0.02 , 4.81)
ER status		
(+) (referent)	76/165	1.00
(-)	31/54	3.73 (0.20 , 68.22)
Eye color		
Light brown	14/31	0.88 (0.23 , 3.41)
Dark brown	43/93	1.28 (0.49 , 3.37)
Black (referent)	45/83	1.00
Skin change during an hour in the summer sun without sunscreen after several months of not being in the sun		
Blistering sunburn	4/11	0.57 (0.08 , 4.26)
Sunburn without blisters	19/43	0.74 (0.12 , 4.72)
Mild sunburn that becomes a tan (referent)	41/93	1.00
Tan with no sunburn	35/49	1.90 (0.26 , 13.87)
No change in skin color	6/16	0.28 (0.03 , 3.22)
Hat or clothes use in the Summer		
Never (referent)	12/30	1.00
Rarely	26/53	2.16 (0.49 , 9.51)
Sometimes	29/40	2.55 (0.54 , 12.13)
Often	19/56	0.60 (0.14 , 2.58)

Always	20/39	1.11 (0.23 , 5.32)
Parity number		
None	2/7	0.58 (0.04 , 8.41)
1 (referent)	14/37	1.00
2	65/128	1.73 (0.53 , 5.63)
3 or more	25/42	2.93 (0.66 , 13.04)
Centre		
1	17/79	1.00
2	41/70	11.63 (2.96 , 45.74)
3	49/70	3.99 (1.24 , 12.79)

[a] All the variables listed above were included in the logistic regression model; [b] Only past smoker was included because no one smoked at enrollment.
